# Establishing a Eukaryotic *Pichia pastoris* Cell-Free Protein Synthesis System

**DOI:** 10.3389/fbioe.2020.00536

**Published:** 2020-06-18

**Authors:** Lingkai Zhang, Wan-Qiu Liu, Jian Li

**Affiliations:** School of Physical Science and Technology, ShanghaiTech University, Shanghai, China

**Keywords:** cell-free protein synthesis, *Pichia pastoris*, yeast, eukaryote, protein expression, cell-free synthetic biology

## Abstract

In recent years, cell-free protein synthesis (CFPS) systems have been used to synthesize proteins, prototype genetic elements, manufacture chemicals, and diagnose diseases. These exciting, novel applications lead to a new wave of interest in the development of new CFPS systems that are derived from prokaryotic and eukaryotic organisms. The eukaryotic *Pichia pastoris* is emerging as a robust chassis host for recombinant protein production. To expand the current CFPS repertoire, we report here the development and optimization of a eukaryotic CFPS system, which is derived from a protease-deficient strain *P. pastoris* SMD1163. By developing a simple crude extract preparation protocol and optimizing CFPS reaction conditions, we were able to achieve superfolder green fluorescent protein (sfGFP) yields of 50.16 ± 7.49 μg/ml in 5 h batch reactions. Our newly developed *P. pastoris* CFPS system fits to the range of the productivity achieved by other eukaryotic CFPS platforms, normally ranging from several to tens of micrograms protein per milliliter in batch mode reactions. Looking forward, we believe that our *P. pastoris* CFPS system will not only expand the CFPS toolbox for synthetic biology applications, but also provide a novel platform for cost-effective, high-yielding production of complex proteins that need post-translational modification and functionalization.

## Introduction

Cell-free protein synthesis (CFPS) systems are emerging as effective platforms for *in vitro* synthetic biology and biotechnology applications from fundamental research to biomanufacturing (Carlson et al., 2012; Bundy et al., 2018; Li et al., 2018b; Swartz, 2018; Khambhati et al., 2019; Liu et al., 2019; Silverman et al., 2020). Such platforms separate the cell growth and the protein synthesis into two stages, which can alleviate the cell's metabolic burden and enhance the productivity. Due to the open nature of CFPS, cell-free reactions can bypass limitations on mass transfer and are more tolerant of toxic protein products. Additionally, the process of CFPS without cell walls can be easily manipulated, controlled, and optimized. Therefore, CFPS systems have recently attracted considerable attention as a robust approach for the production of various proteins, for example, membrane proteins (Henrich et al., 2015; Sonnabend et al., 2017), therapeutic proteins (Min et al., 2016; Wilding et al., 2019), unnatural amino acid modified proteins (Martin et al., 2018; Gao et al., 2019), and difficult-to-express proteins (Li et al., 2016; Jin and Hong, 2018). With the advances of synthetic biology, CFPS technology has also been used to construct protein-based biosensors (Pardee et al., 2016; Thavarajah et al., 2020), metabolic pathways (Goering et al., 2017; Zhuang et al., 2020), high-throughput screening platforms (Sawasaki et al., 2002; Swank et al., 2019), bottom-up synthetic cells (Karzbrun et al., 2014; van Nies et al., 2018), and classroom education kits (Huang et al., 2018; Stark et al., 2018), among others.

Due to the aforementioned emerging applications of CFPS systems, many previous efforts have been focused on the optimization and enhancement of a selected few model systems like the *Escherichia coli* and wheat germ platforms (Carlson et al., 2012; Perez et al., 2016). Unfortunately, these well-developed CFPS systems may have their own disadvantages and drawbacks such as the lack of post-translational modifications (e.g., glycosylation), incorrect protein folding without suitable chaperones, and low protein yields (Zemella et al., 2015). In order to tackle these problems, several new CFPS systems have recently been developed to better mimic the physicochemical environment of native hosts for synthetic biology and biotechnology applications. However, the newly developed CFPS systems are mainly derived from prokaryotic microorganisms, including some from *Streptomyces* species (Li et al., 2017, 2018a; Moore et al., 2017), *Bacillus subtilis* (Kelwick et al., 2016), *Pseudomonas putida* (Wang et al., 2018), and *Vibrio natriegens* (Des Soye et al., 2018; Failmezger et al., 2018; Wiegand et al., 2018). Although a couple of eukaryote-based CFPS systems are available, they are mostly prepared from plant (e.g., wheat germ), insect (e.g., *Spodoptera frugiperda*), and mammalian (e.g., Chinese hamster ovary, CHO) cells (Tarui et al., 2001; Takai et al., 2010; Brödel et al., 2014), which often need laborious and expensive cell extract preparation approaches. For example, it takes 4–5 days to prepare wheat germ extracts (ca. 5 ml) from 5 to 6 kg seeds with the steps of grinding, sieving, extensive washing, and eye selection of the embryo particles (Takai et al., 2010). To date, only a few eukaryotic microorganisms (e.g., *Saccharomyces cerevisiae*), which can be easily cultivated in the laboratory, have been used to develop eukaryotic CFPS platforms (Hodgman and Jewett, 2013; Gan and Jewett, 2014). Despite its success, the protein yield of the *S. cerevisiae*-based CFPS system is relatively low (<10 μg protein/ml) (Hodgman and Jewett, 2013). Therefore, it is highly desirable to develop more eukaryotic microorganism-based CFPS systems to expand the protein expression toolkit for the rapid synthesis, study, and engineering of proteins.

The methylotrophic yeast *Pichia pastoris*, a generally recognized as safe (GRAS) eukaryotic microorganism, has emerged as a reliable and robust chassis host for biotechnological applications in both laboratory and industry (Bill, 2014). Specifically, *P. pastoris* has been well-documented as a cell factory to produce recombinant products such as therapeutic proteins, industrial enzymes, and antimicrobial peptides (Ahmad et al., 2014; Kim et al., 2015; Peña et al., 2018; Yang and Zhang, 2018). The use of *P. pastoris* as an attractive expression system is largely due to its rapid growth on simple media (Darby et al., 2012), readily genetic manipulation tools (e.g., CRISPR-Cas technology) (Raschmanová et al., 2018), and proper eukaryotic post-translational modifications (e.g., humanized *N*-linked glycosylation) (Hamilton et al., 2006). In addition, the genome sequence of *P. pastoris* is available (De Schutter et al., 2009), which provides more opportunities to engineer the organism for desired goals (Peña et al., 2018; Yang and Zhang, 2018). For example, disruption of protease genes in *P. pastoris* generates protease-deficient strains that can prevent recombinant protein degradation and thus increase the product yield (Gleeson et al., 1998; Ni et al., 2008; Wu et al., 2013).

In this work, we aim to establish a eukaryotic microorganism-based CFPS system that is derived from a protease-deficient yeast strain *P. pastoris* SMD1163. After showing the baseline ability to synthesize a reporter protein, we set out to investigate cell lysis procedures to obtain highly active cell extracts, which contain the necessary catalytic components for transcription, translation, and protein folding (e.g., aminoacyl-tRNA synthetases, ribosomes, elongation factors, chaperones, etc.). Then, we assessed the effect of cultivation time, energy conditions, and other physicochemical parameters on protein synthesis yields. Finally, we achieved a ~55-fold increase in protein yields as compared to the initial yield of 0.91 ± 0.12 μg/ml. This work establishes a robust and easy to use eukaryotic CFPS system, which we anticipate that it will serve as an alternative platform for the synthesis of “difficult-to-express” proteins that need, for example, glycosylation, as well as for broad synthetic biology applications.

## Materials and Methods

### Strains and Culture Medium

The protease-deficient yeast strain *P. pastoris* SMD1163 (*pep4 prb1*) was used in this work. Yeast cells were cultivated in a liquid YPD medium consisting of (per liter) 10 g yeast extract, 20 g peptone, and 20 g glucose.

### Plasmid Construction

The superfolder green fluorescent protein (sfGFP) is used as a reporter protein. All plasmids were constructed by modifying the pJL1 expression vector with sfGFP, which is a gift from Michael Jewett (Addgene plasmid # 69496). The cloning was performed according to the Gibson assembly method (Gibson et al., 2009). All gene fragments and sequences used in this study were synthesized by GENEWIZ (Suzhou, China). Initially, a codon optimized sfGFP sequence according to the codon usage in *P. pastoris* was synthesized to replace the original sfGFP gene in pJL1 between the restriction sites *Nde*I and *Sal*I. Then, a synthetic 50 bp poly(A) tail was inserted to the 3′ end of the sfGFP gene. Afterwards, several internal ribosome entry site (IRES) sequences were synthesized and individually cloned to the vector in front of the sfGFP gene. In addition, when the cricket paralysis virus (CrPV) IRES was used, a Kozak sequence (GAAACG) was included after CrPV. All constructs were verified by DNA sequencing (GENEWIZ, Suzhou, China). IRES and codon optimized sfGFP sequences are shown in Table S1.

### Cell Cultivation and Harvest

Yeast cells were cultivated in liquid YPD medium at 30°C in an orbital shaker at 250 rpm. An overnight culture of *P. pastoris* was used to inoculate 1 L fresh YPD medium in a 2.5 L baffled Ultra Yield™ flask (Thomson Instrument Company, USA) with an initial OD_600_ of 0.05. After 18 h cultivation (mid-exponential phase, an OD_600_ of ~6), the cells were harvested by centrifugation at 3,000 g and 4°C for 15 min. Cell pellets were then washed three times with cold washing buffer (30 mM HEPES pH 7.4, 100 mM potassium acetate, 2 mM magnesium acetate, 2 mM dithiolthretol). After the final wash and centrifugation, the pelleted cells were weighed, flash-frozen in liquid nitrogen, and stored at −80°C until further use. Alternatively, cells can be lysed immediately to make cell extracts.

### Cell Extract Preparation

Frozen cells were thawed on ice for 30 min before lysis. The thawed cells were resuspended in 1.5 ml of cold lysis buffer (30 mM HEPES pH 7.4, 100 mM potassium acetate, 2 mM magnesium acetate, 2 mM dithiolthretol, 0.5 mM PMSF) per gram of wet cell weight. Cell disruption was performed using three approaches: sonication, high-pressure homogenization, and 0.5 mm glass beads.

For sonication lysis, the cells were disrupted by using a Q125 Sonicator (Qsonica, Newtown, USA) with 45 s On/60 s Off for five cycles (3 mm diameter probe, 50% of amplitude). For high-pressure homogenization lysis, the smooth suspended cells were lysed by a UH-06 homogenizer (Union-Biotech, Shanghai, China) with two passes at a pressure of 1,200 bar. For glass beads disruption, the cell suspension was mixed with 0.5 mm glass beads (Tansoole, Shanghai, China) in a 50 ml falcon tube at a mass ratio of 1:1 (cell:bead, g/g). Then, the mixture was vortexed vigorously using a vortex mixer (Vortex-Genie 2, New York, USA) for 40 cycles with 1 min on vortex and 1 min on ice.

After cell disruption, the lysate was centrifuged at 30,000 g and 4°C for 30 min. The supernatant was transferred to a fresh tube and clarified again with the same condition. The resultant supernatant was carefully removed and underwent buffer exchange by dialysis with a 3.5 kDa molecular weight cut-off (MWCO) membrane. The lysate was dialyzed against four exchanges of 50-volumes of fresh lysis buffer (30 mM HEPES pH 7.4, 100 mM potassium acetate, 2 mM magnesium acetate, 2 mM dithiolthretol, 0.5 mM PMSF) for 30 min each at 4°C. After dialysis, the extract was centrifuged at 21,000 g and 4°C for 30 min. The resultant supernatant as cell extract was collected, aliquoted, immediately flash-frozen in liquid nitrogen, and finally stored at −80°C until use.

### Cell-Free Protein Synthesis

Coupled cell-free transcription and translation reactions were performed as described previously (Hodgman and Jewett, 2013) with some modifications. Standard CFPS reactions were carried out in 1.5 ml microcentrifuge tubes. Each reaction (15 μl) contains the following components: 25 mM HEPES-KOH pH 7.4, 120 mM potassium glutamate, 6 mM magnesium glutamate, 1.5 mM of each ATP, GTP, CTP, and UTP, 0.1 mM of each of 20 amino acids, 25 mM creatine phosphate, 1.7 mM DTT, 1 mM putrescine, 0.5 mM spermidine, 0.27 mg/ml creatine phosphokinase (from rabbit muscle; Sigma–Aldrich), 16.7 μg/ml plasmid, 60 U T7 RNA polymerase (Thermo Fisher Scientific), and 50% (v/v) cell extract. All reactions were mixed using above conditions and incubated at 23°C for 5 h unless otherwise noted.

### Protein Quantification

The reporter protein sfGFP was used to measure and optimize protein synthesis activity of the *P. pastoris*-based CFPS system. After the reactions, two microliters of the CFPS sample were mixed with 48 μl nuclease-free water and placed in a 96-well plate with flat bottom. The fluorescence of sfGFP was measured using a BioTek SYNETGY H1 plate reader with excitation and emission wavelength at 485 and 528 nm, respectively. sfGFP fluorescence units were converted to concentration (μg/ml) according to a linear standard curve made in house with purified sfGFP. For each protein quantification, at least three independent experiments were carried out using the same cell extract. Then, the protein concentration of each independent reaction was technically measured in triplicate.

## Results and Discussion

### Development of an Initial *P. pastoris*-Based CFPS System

**Figure 1:**
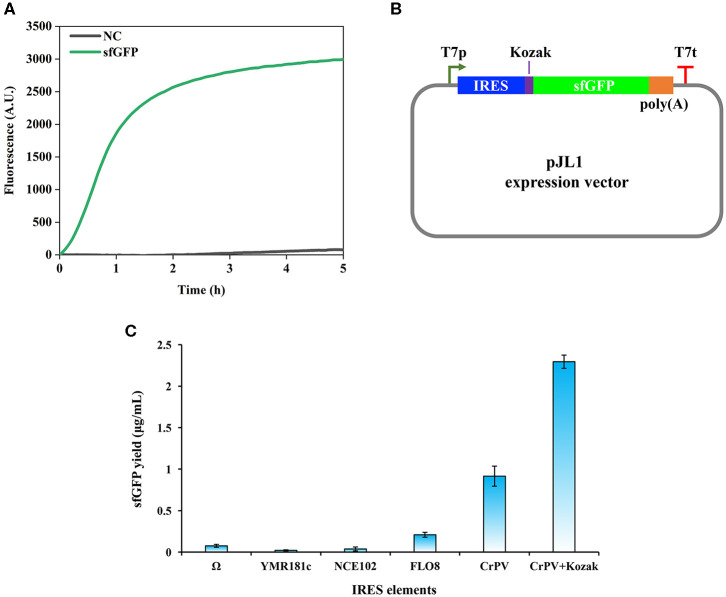
Cell-free protein synthesis of superfolder green fluorescent protein (sfGFP) using *Pichia pastoris* extract. **(A)** Time course of sfGFP synthesis with online fluorescence measurement. NC, negative control without plasmid in the reaction. **(B)** Design of expression vectors based on the pJL1 plasmid. IRES, internal ribosome entry site; Kozak, a 6 bp sequence (GAAACG); poly(A), a 50 bp poly(A) tail; T7p, T7 promoter; T7t, T7 terminator. **(C)** Effects of IRES elements on the cell-free synthesis of sfGFP in a *P. pastoris* CFPS system. Values show means with error bars representing standard deviations (s.d.) of at least three independent experiments.

In general, during the initial development of a new CFPS system, two primary requirements that need to be considered are the choice of a suitable strain and the construction of an efficient expression vector (Brödel et al., 2014; Gan and Jewett, 2014; Kelwick et al., 2016; Li et al., 2017; Des Soye et al., 2018). For example, a protease-deficient *B. subtilis* strain cell extracts produced notably higher and more consistent yields of a reporter protein than a wild-type strain with endogenous proteases (Kelwick et al., 2016). In addition, when constructing an expression vector for eukaryotic CFPS systems, IRES sequences that can recruit eukaryotic ribosomes to initiate cap-independent translation were often investigated to enable combined cell-free transcription and translation (Brödel et al., 2014; Gan and Jewett, 2014). In order to establish a robust *P. pastoris*-based CFPS system, we began our study by trying to adopt the protocol used for the *S. cerevisiae* CFPS system (Hodgman and Jewett, 2013; Gan and Jewett, 2014). Our first step was to find a proper *P. pastoris* strain. Although the strain *P. pastoris* GS115 was reported as a robust host to express recombinant proteins (Gurramkonda et al., 2009; Nie et al., 2014; Zheng et al., 2019; Wang et al., 2020), cell extracts prepared from this strain showed relatively little protein synthesis activity with very low and unreliable yields of sfGFP (data not shown). Then, we switched to the strain *P. pastoris* SMD1163 (*pep4 prb1*), which is derived from GS115 with knocking out of two protease genes (*pep4* encodes protease A and *prb1* encodes protease B) (Gleeson et al., 1998). By doing this, SMD1163 cell extracts were active to synthesize sfGFP. A representative time course of sfGFP synthesis was monitored by online fluorescence measurement (Figure 1A). Our data indicated that the synthesis rate of sfGFP was the highest during the first 1 h reaction with a nearly linear increase manner. Then, the protein synthesis rate declined from 1 to 3 h and no obvious increase of the fluorescence was observed between 3 and 5 h. Therefore, all following CFPS reactions were terminated after 5 h of incubation.

Having validated the combined cell-free transcription and translation, we next set out to evaluate the impact of IRES sequences on the protein synthesis. IRES elements are commonly used by viruses when they infect eukaryotic cells to recruit cellular ribosomes to start cap-independent translation for their own protein synthesis (Baird et al., 2006). This advantage has been taken to develop eukaryotic CFPS systems (Takai et al., 2010; Brödel et al., 2014; Gan and Jewett, 2014), which can facilitate the protein translation process without the laborious preparation of capped mRNA templates. In eukaryotic CFPS systems, two IRES elements often used are the Ω sequence from tobacco mosaic virus (TMV) and the cricket paralysis virus (CrPV) IRES sequence. We, therefore, chose Ω and CrPV IRES sequences to construct our expression vectors. In addition, several IRES sequences have also been identified in *S. cerevisiae* such as *YMR181c, NCE102*, and *FLO8* IRES sequences (Gilbert et al., 2007). Since *P. pastoris* and *S. cerevisiae* are similar yeast strains, the functionality of above three IRES sequences was also evaluated together with Ω and CrPV IRES sequences. The design of expression vectors is shown in Figure 1B. CFPS reactions with different expression vectors were performed in 15 μl batch reactions at 23°C for 5 h. As shown in Figure 1C, the CrPV IRES sequence showed the highest activity among all tested cap-independent translation sequences. The Ω sequence was found to be the best IRES in the *S. cerevisiae* CFPS reaction (Gan and Jewett, 2014), however, it showed low activity in our *P. pastoris* CFPS system. While the three native IRES sequences from *S. cerevisiae* were able to initiate protein translation, their activities were significantly lower than that of the CrPV IRES sequence.

In eukaryotic cells, there are some consensus sequences, which are the so-called Kozak sequences and locate in the upstream of open reading frames (ORF) for ensuring efficient translation initiation (Kozak, 1986, 1987). As reported previously, a Kozak sequence (GAAACG) from the native alcohol oxidase 1 (*AOX1*) gene of *P. pastoris* was often used to construct expression vectors in front of the start codon for enhancing protein translation and recombinant protein yields (Mellitzer et al., 2012; Várnai et al., 2014). The benefit of Kozak sequences was also observed in eukaryotic CFPS systems (Kozak, 1990; Aw and Polizzi, 2019). In order to test if the Kozak sequence (GAAACG) from *AOX1* helps enhance the translation efficiency in our *P. pastoris* CFPS system, it was inserted between the CrPV IRES sequence and the ATG start codon. Our data indicated that the Kozak sequence improved the protein yield to 2.29 ± 0.08 μg/ml, which is about 2.5 times higher than that of the plasmid without the Kozak sequence (the sfGFP yield was 0.91 ± 0.12 μg/ml, Figure 1C). Therefore, the expression plasmid with the CrPV IRES element plus the Kozak sequence was used in our following experiments for further optimization.

### Identification of Optimal Procedures for Preparing *P. pastoris* Cell Extracts

**Figure 2:**
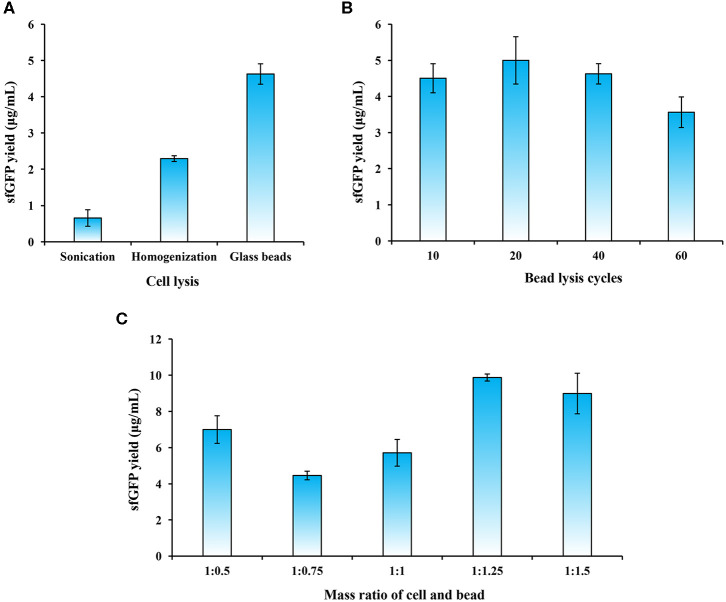
Optimization of cell extract preparation. **(A)** Evaluation of three cell lysis methods. **(B)** Effects of bead lysis cycles on cell extract activity. **(C)** Optimization of the mass ratio of cell and bead (g/g) on sfGFP synthesis. Values show means with error bars representing standard deviations (s.d.) of at least three independent experiments.

Because cell extracts contain essential components like ribosomes, aminoacyl-tRNA synthetases, and chaperons for protein synthesis, it is crucial to prepare highly active cell lysates to support CFPS reactions. For disrupting different types of cells, commonly used cell lysis methods include sonication and high-pressure homogenization (Gan and Jewett, 2014; Kelwick et al., 2016; Li et al., 2017, 2018a; Des Soye et al., 2018; Failmezger et al., 2018; Wang et al., 2018). In addition, glass beads-based disruption has also been used to lyse cells such as *E. coli* (Kigawa, 2010; Sun et al., 2013) and *S. cerevisiae* (Hofbauer et al., 1982; Hodgman and Jewett, 2013) for making cell lysates for CFPS reactions. To identify an optimal cell lysis method, we evaluated the above-mentioned three techniques. Our data demonstrated that the most productive lysate was generated by the bead beating method, which resulted in the highest yield of sfGFP at 4.63 ± 0.28 μg/ml as compared to the other two methods (Figure 2A). The use of beads for cell disruption is simple and promising because no expensive equipment is required like the homogenizer and such method can be easily adopted by other laboratories.

Since the bead lysis method utilizes a grinding mechanism of action, the amount of glass beads in the cell suspension plays an important role in the generation of crushing and grinding forces that break up the cells. We, therefore, next sought to optimize the conditions during the bead lysis process including cell lysis cycles and the mass ratio of glass beads and cell biomass. To this end, we first compared the sfGFP yield with different lysis cycles (one cycle is 1 min on vortex mixer and 1 min on ice for cooling). The results indicated that the highest sfGFP yield reached at 4.99 ± 0.66 μg/mL with 20 cycles of cell lysis (Figure 2B). However, more cycles (>40) slightly reduced the protein yields. Next, we investigated the effect of mass ratio of glass beads and cell biomass on the sfGFP synthesis. The data suggested that a higher ratio of bead in the mixture was better to grind the cells with a ratio of 1:1.25 (cell:bead, g/g) maximizing protein expression in our experiments (Figure 2C). This is likely due to the fact that more beads may provide higher grinding forces and thus can efficiently disrupt cells as reported previously (Hofbauer et al., 1982; Kigawa, 2010; Sun et al., 2013).

### Effect of Cell Harvest Time on CFPS Reactions

**Figure 3:**
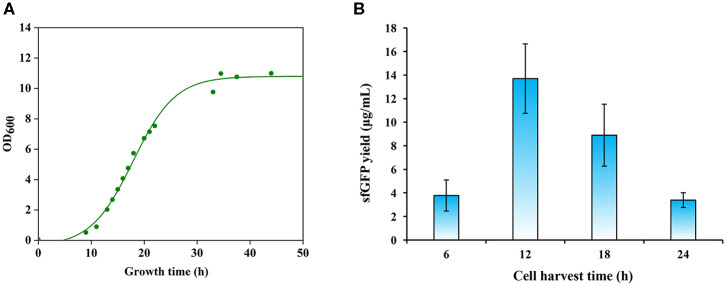
Effects of cell growth phase on protein synthesis. **(A)** A representative growth curve of *Pichia pastoris*. **(B)** Comparison of sfGFP synthesis with cell extracts prepared from different growth phases. Values show means with error bars representing standard deviations (s.d.) of at least three independent experiments.

The activity of crude extract-based CFPS reactions is essentially dependent on the composition of the cellular machinery (e.g., ribosomes) at the time of cell harvest. Previous studies have shown that cells harvested during the exponential growth phase, especially, in the mid-exponential phase, could provide benefits for protein synthesis because the translation machinery is most active (Hodgman and Jewett, 2013; Li et al., 2017; Wang et al., 2018). We were curious to determine the optimal cell harvest time for *P. pastoris* to prepare highly active cell extracts. We grew *P. pastoris* cells in 1 L media with an initial OD_600_ of 0.05 for 45 h at 30°C and 250 rpm. According to the cell growth curve (Figure 3A), we harvested cells after the cultivation at 12, 18, and 24 h, which spanned a range of early to late exponential growth phase in our culture media. Then, we prepared cell extracts from each of these cultivations. To compare these extracts, we performed CFPS reactions in parallel and sfGFP yields were determined. We found that cell extracts prepared from the early exponential phase (12 h) with a final OD_600_ of ~2 were most active and synthesized the highest yield of sfGFP at 13.70 ± 2.94 μg/ml (Figure 3B). In addition, we harvested cells before the early exponential phase after 6 h cultivation for comparison. However, the protein yield from such extracts was significantly lower than the highest yield (Figure 3B). Not like other CFPS systems, cell extracts derived from the early exponential phase rather than the mid-exponential phase favored protein synthesis in our *P. pastoris* CFPS system. Taken together, our results highlight the importance of cell harvest as a critical factor for development and optimization of CFPS systems.

### Optimization of CFPS Reaction Conditions

**Figure 4:**
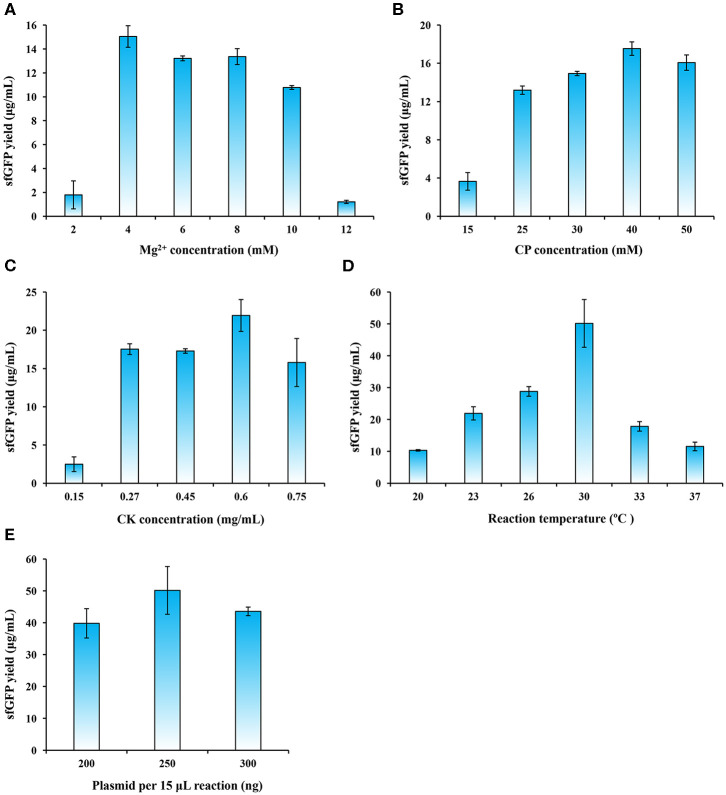
CFPS reaction optimization for enhancing sfGFP expression yields. The CFPS reaction was optimized by altering **(A)** Mg^2+^ concentration, **(B)** CP (creatine phosphate) concentration, **(C)** CK (creatine phosphokinase) concentration, **(D)** reaction temperature, and **(E)** plasmid concentration for sfGFP synthesis. Values show means with error bars representing standard deviations (s.d.) of at least three independent experiments.

In order to further increase protein expression yields, we next carried out a systematic optimization of the *P. pastoris* CFPS system. Specifically, we investigated the effects of magnesium ion concentration, energy regeneration, template plasmid concentration, and reaction temperature on protein yields. We started with Mg^2+^ concentration, which is known to be a critical cation used in cell-free systems that influences the assembly and activity of ribosomes (Klein et al., 2004; Yamamoto et al., 2010; Petrov et al., 2012). Previous reports showed that different CFPS systems also need different concentrations of Mg^2+^ ions and the optimal values have to be optimized accordingly (Brödel et al., 2014; Kelwick et al., 2016; Li et al., 2017; Des Soye et al., 2018; Wang et al., 2018). We performed CFPS reactions with a range of Mg^2+^ concentrations from 2 to 12 mM. Our results suggested that 4 mM gave rise to the highest sfGFP yield of 15.04 ± 0.90 μg/ml (Figure 4A). Similar yields were obtained at around 13 μg/ml with 6 and 8 mM Mg^2+^ concentrations, respectively.

Energy supply in CFPS systems is an important factor that affects the efficiency of protein synthesis (Kim and Swartz, 1999; Jewett et al., 2008). The energy regeneration system with the pair of creatine phosphate/creatine phosphokinase (CP/CK) is often used in eukaryotic CFPS systems (Tarui et al., 2001; Takai et al., 2010; Hodgman and Jewett, 2013; Brödel et al., 2014). The CP/CK energy system was also employed in our *P. pastoris* CFPS system. Therefore, we next sought to optimize each concentration of CP and CK in the reaction mixture. To do this, we varied the concentrations of CP and CK separately in CFPS reactions. We observed that the optimal concentration for CP and CK were 40 mM and 0.6 mg/ml (Figures 4B,C), respectively. The optimized CP/CK system improved the sfGFP yield to 21.94 ± 2.08 μg/ml, which is ~70% higher than the yield without optimization.

Another key factor needs to be optimized is the reaction temperature, because it affects protein synthesis and protein folding (Gan and Jewett, 2014; Li et al., 2017; Wang et al., 2018). We run CFPS reactions with different temperatures ranging from 20 to 37°C. Our data indicated that the sfGFP yields gradually improved with the increase of temperature from 20 to 30°C (Figure 4D). However, the yields notably decreased at temperatures of 33 and 37°C. The reaction temperature at 30°C, which is the same as the preferred growth temperature of *P. pastoris*, remarkably favored protein synthesis with the highest yield of 50.16 ± 7.49 μg/ml that is >2 times higher than the yield at 23°C (Figure 4D). Our finding is similar to other CFPS systems like *E. coli* and *B. subtilis* at 30°C (Kelwick et al., 2016; Li et al., 2016), albeit the optimal temperature for the *S. cerevisiae* CFPS reaction is 24°C (Gan and Jewett, 2014).

Finally, we investigated the impact of plasmid concentration on the performance of our *P. pastoris* CFPS system. The reactions were carried out in 15 μl volume by adding 200, 250, or 300 ng of template plasmids. The results showed that the sfGFP expression peaked when 250 ng of plasmid was supplied as template (Figure 4E). Taken together, the final, optimized *P. pastoris* CFPS system described here is capable of synthesizing ~50 μg/ml of sfGFP in a 5 h batch reaction, which is a ~55-fold increase in protein yields as compared to the initial yield of 0.91 ± 0.12 μg/ml. Our yield fits to the range of the yields achieved by other eukaryotic CFPS platforms, normally ranging from several to tens of micrograms protein per milliliter in batch mode reactions (Carlson et al., 2012). Such CFPS platforms include, for example, the insect system (25 μg/ml) (Tarui et al., 2001), the CHO system (~50 μg/ml) (Brödel et al., 2014), and the *S. cerevisiae* system (<10 μg/ml) (Hodgman and Jewett, 2013). While we successfully established the *P. pastoris*-based CFPS platform, more efforts are still needed in the future to further improve the overall protein yields through upstream and downstream engineering strategies, for example, strain engineering (e.g., using nuclease-deficient strains), bioprocess engineering (e.g., high cell density cultivation in bioreactors), and reaction engineering (e.g., semi-continuous and continuous reaction formats). In addition, a recent report has demonstrated that enhancing ribosome content during cell growth could provide more active cell extracts, giving rise to higher protein yields in a CFPS system (Aw and Polizzi, 2019). Therefore, combining this strategy with above-mentioned solutions might further enhance the productivity of the *P. pastoris* CFPS system.

Having obtained an optimized CFPS system, we sought to demonstrate the utility of the platform. We chose two other eukaryotic proteins, luciferase and human serum albumin (HSA, a therapeutic protein), as our targets, which were expressed and analyzed by western blotting. The results indicated that both proteins were also successfully expressed and more than 90% of total proteins were soluble (Figure S1). As a result, the ability to use the newly developed *P. pastoris* CFPS system to express different proteins demonstrated its potential applications for the synthesis of more complex eukaryotic proteins such as membrane and glycosylated proteins.

## Conclusions

In this study, we developed a robust and easy-to-use yeast-based CFPS system, which is derived from the protease-deficient strain *P. pastoris* SMD1163. In particular, we designed a suitable expression vector, identified a simple cell extract preparation protocol, and performed a physicochemical optimization. After the above systematic optimization, we were able to achieve sfGFP yields of ~50 μg/ml in 5 h batch reactions. The high-yielding CFPS capacity, together with the heterologous protein expression capability of *P. pastoris*, makes it a valuable addition to the current existing CFPS platforms. Looking forward, we believe that this work will not only provide an alternative eukaryotic CFPS platform for the synthesis of “difficult-to-express” proteins like glycoproteins, but also set the stage for exciting, novel applications in biotechnology and synthetic biology.

## Data Availability Statement

All datasets generated for this study are included in the article/Supplementary Material.

## Author Contributions

LZ, W-QL, and JL designed the experiments. LZ performed the experiments. LZ and JL analyzed the data and prepared the figures. JL conceived the project, supervised the research, and wrote the manuscript. All authors contributed to the article and approved the submitted version.

## Conflict of Interest

The authors declare that the research was conducted in the absence of any commercial or financial relationships that could be construed as a potential conflict of interest.
