# Supplementary material for: Establishing a Eukaryotic Pichia pastoris Cell-Free Protein Synthesis System
Source: Front Bioeng Biotechnol. 2020 Jun 18;8:536. doi: 10.3389/fbioe.2020.00536 (PMC7314905; doi:10.3389/fbioe.2020.00536)
Supplement: Supplementary file 1 [file Data_Sheet_1.PDF]

Supplementary Material

Establishing a Eukaryotic *Pichia pastoris* Cell-Free Protein Synthesis System

Lingkai Zhang, Wan-Qiu Liu and Jian Li*

School of Physical Science and Technology, ShanghaiTech University, Shanghai, China

*Correspondence:
Jian Li
lijian@shanghaitech.edu.cn

Supplementary Table S1. List of internal ribosome entry site (IRES) sequences used in this study and the codon-optimized sfGFP sequence.

Name	Sequence
Ω	TATTTTTACAACAATTACCAACAACAACAAACAACAAACAACATTACAAT<br>TACTATTTACAATTACA
CrPV	AAAGCAAAAATGTGATCTTGCTTGTAAATACAATTTTGAGAGGTTAATAA<br>ATTACAAGTAGTGCTATTTTTGTATTTAGGTTAGCTATTTAGCTTTACGTT<br>CCAGGATGCCTAGTGGCAGCCCCACAATATCCAGGAAGCCCTCTCTGCGG<br>TTTTTCAGATTAGGTAGTCGAAAAACCTAAGAAATTTACCTGCT
YMR181c	CCCTTATATTTACTCACCTTCCCCCTACTCTAATTCTCCTGATTTCAGAAC<br>AAAAAAAAA
NCE102	CGCCCCCTCACAGAAACTTATGGGCAGCTTGCTGCCTTAACGGAATTGAC<br>TAGAATTGGTTTGATTTTTTTTTCTTCCTACTTCTTCTTCCATTTCCCTCCCC<br>TCTTCCTCATATATGTATACAGAAAAATCATACCCCTATAAATTCCTTGGC<br>CCCAATCTTCTGTCAGATTTTCCTTTATAAAAGAGTCTTTGTTTTGTAATT<br>AAAAGATACTTTTTTCTTTCTTCTTCTACGTCTCCTTTTTTTTTTTTAAGAA<br>AATTTAACTTATACCACTATTTTGTTCGCAATTGATCAAGAAAAAATACA<br>ATTGAAAAGGTTTTACATTTTTAATTTTTCTGCTCATCGCGCTTTTTTAAA<br>AGGATAAATAAACATTTCTTTAAAAAACATCTTCAATAAGAAAAATCGGT<br>TAAAAAAACTTTTCTTCTCAAAGCATACCTAATAACAATATAATCCCATA
FLO8	ATTGAAAAAGTGACCATTTTTTACTCCTGTTCAAGCGCATTTGCTTTGATA<br>CCATTTTGTTTGCCGAAGACACGGTGAGTTGACGTTAGTAAGTCACTGAG<br>GCTATAAAAAATAAACACGAAGACGTTTATAGACATAAATAAAGAGGAA<br>ACGCATTCCGTGGTAGA
sfGFP	ATGTCTAAGGGAGAGGAGTTGTTCACCGGTGTTGTCCCAATCTTGGTTGA<br>GTTGGACGGTGACGTTAACGGTCACAAGTTCTCCGTCAGAGGTGAGGGTG<br>AAGGTGACGCCACCATCGGTAAGTTGACCTTGAAGTTTATCTGTACCACT<br>GGTAAGTTGCCCGTTCCATGGCCAACCTTGGTTACCACTTTGACCTACGGT<br>GTCCAGTGCTTCTCCAGATACCCAGACCACATGAAGCGTCACGATTTCTT<br>CAAGTCCGCCATGCCAGAGGGTTACGTTCAAGAAAGAACTATTTCTTTCA<br>AGGACGATGGTAAGTATAAAACCAGAGCTGTTGTCAAGTTCGAGGGTGA<br>CACCTTGGTCAACAGAATCGAGTTGAAGGGTACTGATTTTAAGGAAGATG<br>GAAACATTTTGGGTCATAAGTTGGAATATAACTTCAACTCCCATAACGTC<br>TACATCACCGCCGACAAGCAGAAGAACGGTATCAAGGCCAACTTCACCG<br>TTCGTCACAACGTTGAGGATGGTTCCGTTCAGTTGGCTGACCACTACCAA<br>CAGAACACCCCAATTGGTGACGGACCAGTCTTGTTGCCAGACAACCACTA<br>CTTGTCCACCCAAACCGTCTTGTCCAAGGACCCAAACGAGAAGGGTACCA<br>GAGACCACATGGTCTTGCACGAATACGTCAACGCTGCCGGAATCACTTGG<br>TCTCATCCACAATTCGAAAAGTAA

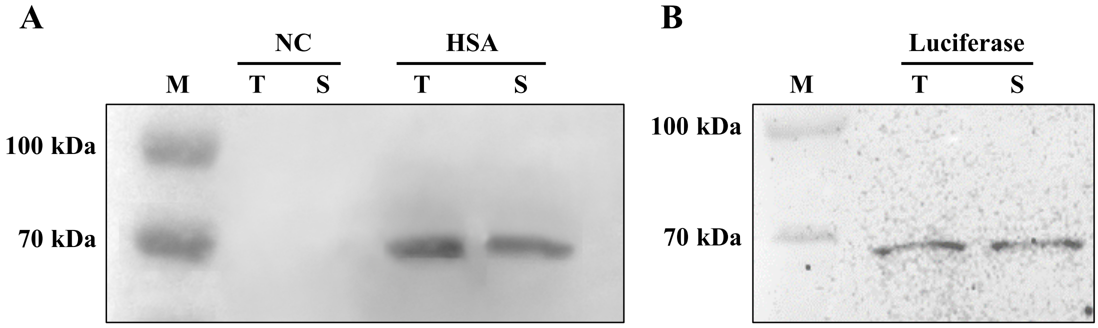

Figure S1 Western blotting analysis of cell-free expressed **(A)** human serum albumin (HSA, 70.2 kDa) and **(B)** luciferase (61.6 kDa). M, protein molecular weight marker; NC, negative control without plasmid in the reaction; T, total protein; S, soluble protein.
